# Author Correction: Exciton-driven change of phonon modes causes strong temperature dependent bandgap shift in nanoclusters

**DOI:** 10.1038/s41467-025-58988-9

**Published:** 2025-04-28

**Authors:** Franziska Münzer, Severin Lorenz, Jiwoong Yang, Taufik Adi Nugraha, Emilio Scalise, Taeghwan Hyeon, Stefan Wippermann, Gerd Bacher

**Affiliations:** 1 Werkstoffe der Elektrotechnik and CENIDE, Universität Duisburg-Essen, Bismarckstraße 81, 47057 Duisburg, Germany; 2 Center for Nanoparticle Research, Institute for Basic Science (IBS), Seoul, 08826 Republic of Korea; 3 Department of Energy Science & Engineering, Daegu Gyeongbuk Institute of Science & Technology (DGIST), Daegu, 42988 Republic of Korea; 4 Max-Planck-Institut für Eisenforschung, Max-Planck-Strasse 1, 40237 Düsseldorf, Germany; 5 School of Chemical and Biological Engineering, and Institute of Chemical Processes, Seoul National University, Seoul, 08826 Republic of Korea

**Keywords:** Electronic materials, Quantum dots, Electronic properties and materials

Correction to: *Nature Communications* 10.1038/s41467-020-17563-0, published online 17 August 2020

Since the publication of this work, Franziska Münzer has changed their name from Franziska Muckel. This has now been amended in the HTML and PDF versions of the article. The original article has been corrected.

