# Supplementary material for: Exciton-driven change of phonon modes causes strong temperature dependent bandgap shift in nanoclusters
Source: Nat Commun. 2020 Aug 17;11:4127. doi: 10.1038/s41467-020-17563-0 (PMC7431586; doi:10.1038/s41467-020-17563-0)
Supplement: Supplementary file 1 — Supplementary Information [file 41467_2020_17563_MOESM1_ESM.pdf]

Supplementary Information

Exciton-driven change of phonon modes causes strong temperature dependent bandgap shift in nanoclusters

Franziska Muckel[1], Severin Lorenz[1], Jiwoong Yang[2,3], Taufik Adi Nugraha[4], Emilio Scalise[4], Taeghwan Hyeon[2,5], Stefan Wippermann[4], and Gerd Bacher[1]

[1] Werkstoffe der Elektrotechnik and CENIDE, Universität Duisburg-Essen, Bismarckstraße 81, 47057 Duisburg, Germany
[2] Center for Nanoparticle Research, Institute for Basic Science (IBS), Seoul 08826, Republic of Korea
[3] Department of Energy Science & Engineering, Daegu Gyeongbuk Institute of Science & Technology (DGIST), Daegu 42988, Republic of Korea
[4]Max-Planck-Institut für Eisenforschung, Max-Planck-Strasse 1, 40237 Düsseldorf, Germany
[5] School of Chemical and Biological Engineering, and Institute of Chemical Processes, Seoul National University, Seoul 08826, Republic of Korea

Supplementary Figures

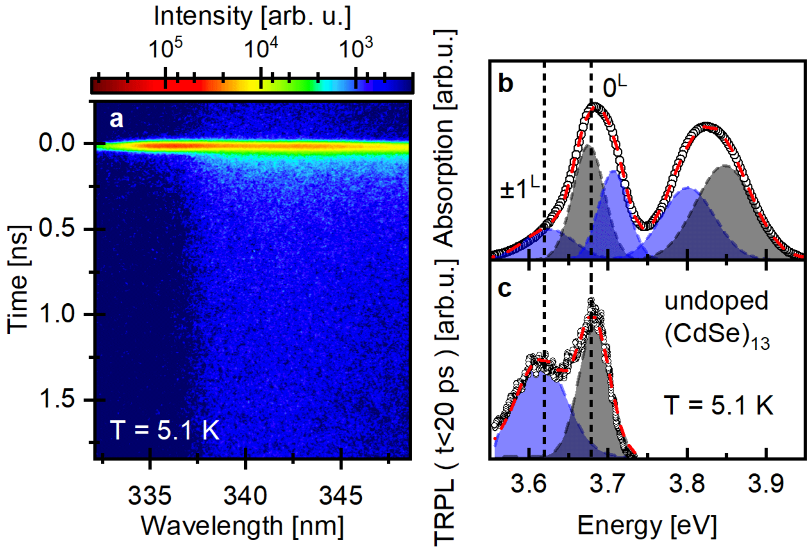

Supplementary Figure 1 | Time resolved photoluminescence of undoped $(CdSe)_{13}$ MSCs. a, Streak image of the transient PL from undoped $(CdSe)_{13}$ MSCs at 5 K for the first 1.8 ns after 100 fs laser excitation. For the evaluation of the temperature dependent bandgap shift, the initial component of the PL signal directly after pulsed laser excitation has been extracted integrating from 0 ps to 20 ps (depicted in panel c). Comparison of the absorption **(b)** and time-resolved PL spectra within the first few 20 ps after pulsed laser excitation **(c)** of undoped $(CdSe)_{13}$ MSCs reveals the coincidence with the fine structure transitions, which we assign in first approximation according to the notation of Efros et al.[1]. Virtually no Stokes shift of the individual fine structure transitions between absorption and emission is observed within our experimental resolution (1 nm, corresponding to about 10 meV). Positions of the two energetically lowest transitions are marked with dashed black lines. Absorption and emission are fitted with two and five Gaussian peaks, respectively, magneto-optically active (inactive) peaks are shown in blue (grey) and the sum as dashed red line, applying identical widths and peak positions within our resolution. The magneto-optical activity of the individual transitions is derived from MCD experiments.[2]

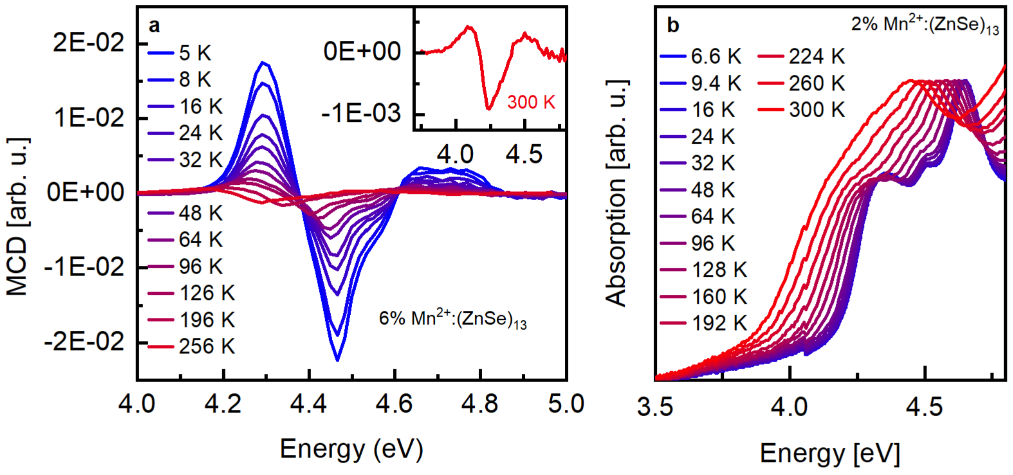

Supplementary Figure 2 | Temperature dependent MCD and absorption spectra of Mn^{2+}-doped $(ZnSe)_{13}$ a, Temperature dependent MCD signal of Mn^{2+}-doped $(ZnSe)_{13}$ MSC. Similar to MSC consisting of CdSe, Mn^{2+}:$(ZnSe)_{13}$ MSCs with the same number of atoms exhibit a similiarly enhanced temperature dependent shift of the bandgap compared to bulk ZnSe. **B** Temperature dependence of the absorption spectra of 2% Mn^{2+}-doped $(ZnSe)_{13}$.

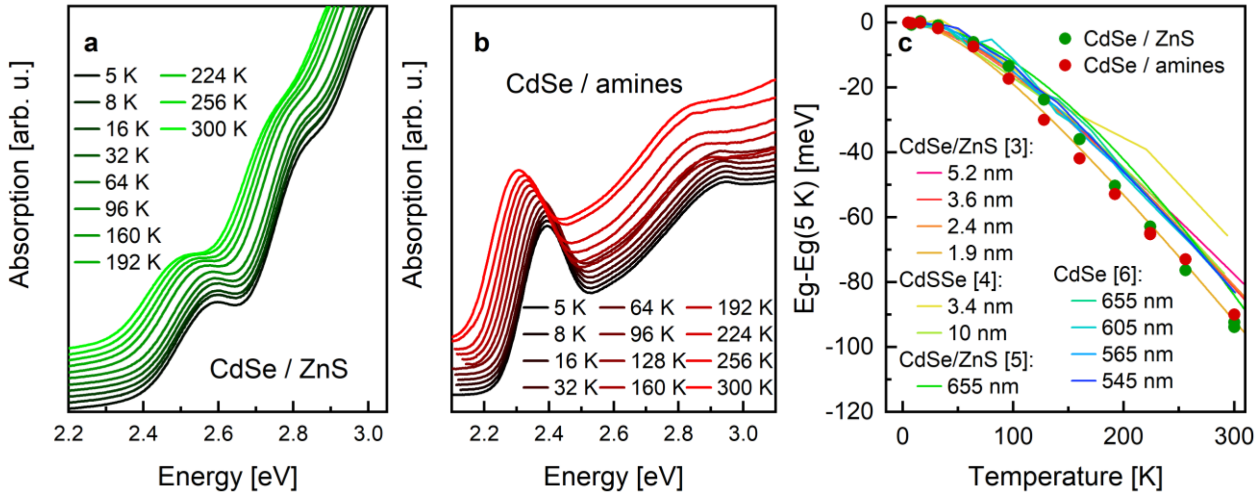

Supplementary Figure 3 | Temperature dependence of the bandgap in CdSe QDs. Absorption of conventional CdSe QDs with ZnS shell (**a**) or capped by amine ligands (**b**) for different temperatures. Spectra are shifted vertically for clarity. **c**, Comparison of the energy shift for the first absorption peak with temperature as extracted from Gaussian fits from panel (**a**) and (**b**) with temperature depended bandgap shifts for CdSe quantum dots reported in literature. [3–6]

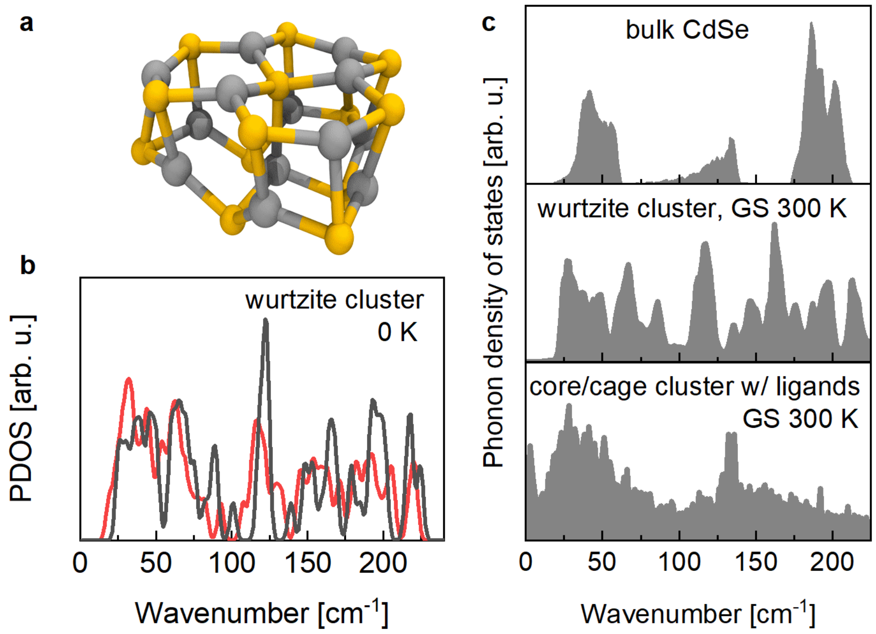

Supplementary Figure 4 | PDOS of the sliced-wurtzite (a) and core/cage (b) models at different temperatures. Note, that for the core/cage cluster with MA ligands the PDOS contains in addition to modes inherent to the CdSe cluster vibrational and rotational modes stemming from the MA ligands, which fill up the phonon gap towards zero energy. **a,** Crystal structure of the bare sliced-wurtzite MSC. **b,** Ground (grey) and excited (red) state PDOS of the wurtzite cluster at 0 K. **c,** Comparison of the 300 K PDOS of the sliced-wurtzite and the core-cage cluster to the PDOS of bulk CdSe[7]. Both types of clusters exhibit significantly changed PDOS compared to the bulk material. In order to compare the acoustical phonon modes quantitatively, we determined the mean wavenumber taking into account modes up to 75 cm^{-1}. While there is virtually no shift between bulk (44.7 cm^{-1}) and the sliced-wurtzite structure (46.3 cm^{-1}), the mean phonon energy is significantly smaller in the core/cage cluster (37.0 cm^{-1}). Note that the arrangement of the atoms in the sliced-wurtzite model is much closer to that in bulk CdSe compared to that of the core/cage model.

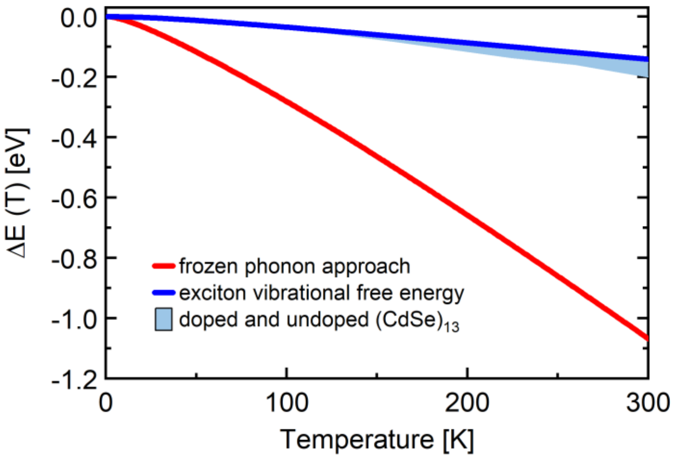

Supplementary Figure 5 | Frozen Phonon approach. Comparison of the bandgap shift with temperature, calculated using the frozen phonon approach (red) and the change in vibrational free energy (blue), both in harmonic approximation. The ground state phonons at 0 K (compare grey trace in Supplementary Figure 4b)), which enter the exciton free energy calculation, are the same ones that are used in the frozen-phonon approach. The range of experimental data is highlighted as a light blue area. The zero point motion renormalization energy calculated via the frozen phonon approach is 397 meV, while it accounts for 56 meV if calculated using the change in vibrational free energy for an exciton.

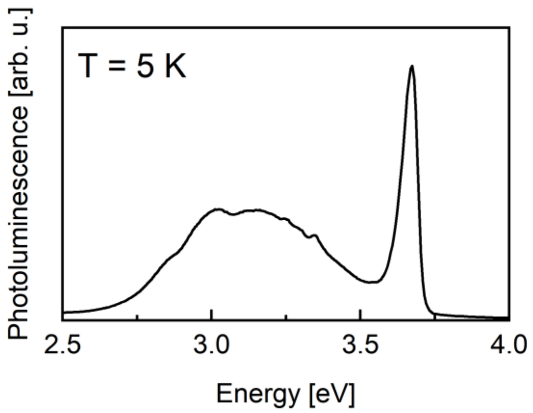

Supplementary Figure 6 | Full time-integrated PL spectrum of undoped cluster at 5 K and an excitation power of 30 µW. Besides the band edge transition at 3.65 eV, the spectrum depicts a broad luminescence around 3.2 eV, which we hypothesize to result from the (HOMO→LUMO) transition, with the LUMO describing a mid-gap state stemming from the surface. It has been frequently reported[8–11] that in small CdSe nanoclusters the surface tends to reorganize in a way that a mid-gap state is formed (referred to as LUMO in our work). This state is observed to be weakly absorbing, but more prominent in photoluminescence[8–11], depended on temperature and quality of the sample. However, at lower excitation intensities and monitoring the transient PL, we are able to separate the band edge emission around 3.65 eV from the mid-gap state related emission around 3.2 eV

Supplementary Tables

Supplementary Table 1 | Statistics on the anomalous bandgap shift in undoped and Mn^{2+}-doped $(CdSe)_{13}$ MSC. The table summarizes the bandgap shifts between cryogenic and room temperature for undoped and Mn^{2+}-doped $(CdSe)_{13}$ MSCs with different doping concentrations. In bulk $\frac{dE_g}{dT}$ accounts for -0.313 meV/K and -0.375 meV/K for cubic and wurtzite CdSe, respectively[12].

Experimental approach	Mn^{2+} concentration	$\Delta E_g(5\ K \rightarrow 300\ K)$ [meV]	$\frac{dE_g}{dT}\ (160\ K \rightarrow 300\ K)\ [meV/K]$
MCD	2%	195	-0.880
	4%	-	-0.609
	6%	162	-0.740
	8%	150	-0.638
	10%	156	-0.644
PLE	2%	200	-0.827
	4%	160	-0.636
	6%	165	-0.683
Absorption	0%	152	-0.698
	2%	189	-0.858
	6%	164	-0.709
	8%	169	-0.627
	10%	156	-0.741
TRPL	0%	163	-0.626
Mean		168 ± 16	-0.709 ± 0.09

Supplementary Table 2 | Phonon frequencies, ω_i, obtained from the force constant matrix at T = 0 K for the sliced-wurtzite $(CdSe)_{13}$ MSCs in the ground (gs) and HOMO → LUMO+1 (exc) electronic configuration, respectively.

phonon modes [cm^{-1}]		phonon modes [cm^{-1}]		phonon modes [cm^{-1}]	
exc	gs	exc	gs	exc	gs
256.095064	224.502221	172.535078	179.04898	114.826075	118.95078
248.902095	224.308943	169.904243	178.63693	112.873484	118.49612
221.477328	218.357003	164.587471	168.34946	107.548953	117.51214
219.976789	218.178223	162.458072	168.32442	92.756873	100.8512
217.14754	217.425993	159.605916	165.78753	82.355669	89.381238
206.489571	215.339866	149.397693	153.43399	77.913707	89.228991
204.981464	201.184041	145.988757	153.15816	73.593562	86.852253
201.554726	201.129968	144.078192	148.45185	69.625286	82.228448
197.153618	198.387655	133.479572	147.97574	68.519022	75.355318
192.981298	197.96228	130.138456	139.10403	64.834253	74.975455
192.749362	196.02975	126.894772	124.06561	64.645238	70.386623
189.154293	193.76597	122.213956	123.1968	63.460778	70.111788
187.654677	193.54481	121.412544	122.6393	61.362876	69.213983
183.39972	191.5514	118.743711	122.61158	60.377826	66.080541
182.141025	191.35895	117.867973	122.28847	59.536511	65.541318
178.924408	184.63216	115.730709	121.71341	56.472868	65.302316

phonon modes [cm^{-1}] exc	gs
54.277805	62.201347
53.243414	62.030614
50.633465	59.568474
46.810307	59.447379
45.448582	51.098566
44.168083	49.673785
43.162327	48.895816
41.344244	46.054828
39.214273	45.776477
37.331445	44.648767
34.969491	41.14315
33.959556	39.789086
32.98467	39.221207
32.005578	36.274854
30.478023	36.117087
29.677912	34.147212
28.241249	30.678114
26.661259	30.589346
24.921599	27.711511
22.574215	25.305989
18.866776	25.144288

Supplementary Table 3 | Phonon frequencies ω_i obtained from the force constant matrix at T = 0 K for core/cage $(CdSe)_{13}$ MSCs terminated by methylamine ligands in the ground (gs) and HOMO → LUMO+1 (exc) electronic configurations, respectively.

phonon modes [cm^{-1}] exc	gs
4917.086	4917.482
4902.794	4900.228
4875.716	4896.006
4850.533	4888.126
4820.373	4814.917
4793.448	4791.906
4363.725	4363.654
4342.545	4344.064
4319.458	4341.583
4310.814	4339.67
4256.424	4252.616
4232.462	4220.158
2905.421	2905.859
2899.339	2899.435
2898.083	2899.17
2893.031	2898.309
2889.985	2891.652
2886.593	2889.981
2881.613	2882.334
2861.13	2859.496
2858.014	2859.028
2856.028	2855.527
2853.021	2854.317
2704.337	2703.568
2692.315	2692.836
2682.499	2691.507
2679.723	2688.484
2673.282	2686.502
2631.806	2657.775
1933.187	1942.958
1928.86	1924.947
1925.244	1924.207
1922.341	1919.872
1913.511	1915.58
1893.095	1898.213
1592.647	1593.476
1555.685	1553.96
1548.222	1553.815
1547.895	1550.23
1543.976	1549.797
1541.695	1544.386
1417.775	1418.456
1372.147	1406.418
1347.928	1347.873
1346.148	1347.051
1341.44	1343.969
1339.621	1342.654
1278.446	1280.994
1273.371	1279.279
1272.238	1278.778
1271.15	1272.618
1270.87	1271.846
1270.412	1271.236
1103.625	1212.857
1102.192	1105.17
1098.959	1102.437
1096.301	1100.796
1068.426	1094.836
1065.781	1067.852
1063.153	1066.869
1048.688	1051.442
1040.923	1044.64
1031.626	1042.968
1030.812	1033.605
1030.612	1030.886
1028.406	1030.539
998.0087	1029.42
805.2596	798.69
793.5433	798.2444
765.6271	757.1834
754.7217	756.8251
737.2962	727.8144
717.0289	666.6891
664.0124	656.2143
656.0319	649.4704
649.1341	646.4048
644.586	636.8776
602.8328	598.3164
576.8052	575.5802
565.1419	560.3887
552.8316	514.9221
507.7987	510.2422
492.229	469.5899
463.2581	463.9708
453.6906	459.0535
450.3964	458.067
443.4798	444.0568
422.141	436.4758
421.3641	425.3413
420.9622	422.1694
420.9492	421.548
417.8965	421.4844
414.9934	421.2899
408.5879	421.0116
406.125	415.07
400.0712	410.2633
396.1395	408.5244
392.9975	402.3732
386.5761	395.803
380.525	384.7047
378.9704	383.0613
376.4925	380.7904
362.641	375.3275
358.3583	372.8275
355.6146	371.0777
349.9619	369.0349
347.2591	363.0468
346.3735	351.079
341.3574	349.81
337.7621	345.0788
333.2717	343.4691
332.147	338.8472
328.2695	337.1126
326.9265	335.9276

phonon modes [cm^{-1}]	
**exc**	**gs**
319.7944	313.569
314.3619	309.9325
310.2026	305.1712
301.4037	303.7448
297.2122	298.7244
295.5819	295.3403
290.8802	289.0628
288.4431	284.9198
284.1897	282.2184
279.9415	279.5211
279.5405	276.4731
269.4286	275.5159
266.1859	268.1959
260.2999	261.8238
242.5343	258.4116
230.052	254.0444
191.4847	231.0098
176.3311	191.3319
163.1696	189.4201
137.4076	136.9944
128.5692	132.6007
127.3408	131.1936
125.3944	128.0595
113.9536	114.0097
106.6336	110.4344
106.2555	107.7233
99.22295	103.5453
96.97347	101.3595
92.19228	99.42281

phonon modes [cm^{-1}]	
**exc**	**gs**
88.64347	95.53406
87.48708	93.51163
85.89359	91.36862
84.29256	88.39464
79.58671	86.8366
78.53596	85.19904
77.29349	83.95153
75.05521	81.34069
72.18522	77.68302
71.3383	77.34097
69.36704	77.14911
66.26546	74.25019
63.31922	73.22499
61.88139	69.70531
61.27862	68.41973
58.74797	66.17718
56.54024	63.1967
55.64826	61.59068
54.20511	58.42306
51.96922	57.03823
50.99202	56.78021
49.58063	55.14018
48.04871	54.25024
46.85095	52.06593
45.05498	49.63545
44.00772	49.07623
41.72072	48.70915
40.37592	44.64877
38.88699	43.08035

phonon modes [cm^{-1}]	
**exc**	**gs**
37.82353	41.72724
36.22234	40.1666
35.56704	38.65554
33.58515	36.82541
33.21061	35.41764
30.11451	35.10144
29.09026	33.12863
27.89733	31.77107
27.11635	28.43795
25.62633	22.87338
25.01961	21.57014
19.31545	20.66883
18.92434	20.25688
17.64536	18.1243
17.42828	16.28284
13.81645	14.45135
13.16126	14.06032
12.76266	12.86875
12.0616	12.53695
11.38904	12.1738
10.17865	11.76486
10.1385	11.18425
9.755777	10.98802
8.29446	10.8134
7.628423	10.01708
7.356215	6.996231
4.455427	5.736151

Supplementary Discussion

Supplementary Discussion 1 | Influence of the quantum confinement on the temperature dependence of the band gap in $(CdSe)_{13}$.

While in bulk semiconductors the band gap shrinkage with temperature is widely accepted to be a cumulative effect of the thermal lattice expansion and electron-phonon interactions[13], in strongly confined MSCs additional impacts have to be considered including changes in the Coulomb or confinement energy with temperature (in the absence of a shell any strain induced changes can be neglected)[4,6,14]:

$$\frac{\mathrm{d}E_g}{\mathrm{d}T} = \left(\frac{\delta E_g}{\delta T}\right)_{lattice} + \left(\frac{\delta E_g}{\delta T}\right)_{phonon} + \left(\frac{\delta E_g}{\delta T}\right)_{Coulomb} + \left(\frac{\delta E_g}{\delta T}\right)_{confinement} \quad (1)$$

First, the confinement energy (approx. 1.8 eV in the clusters) changes due to the lattice dilatation, which, considering the bulk lattice expansion coefficient at room temperature

(between $2.5\cdot10^{-6}$ K^{-1} and $4.3\cdot10^{-6}$ K^{-1}) [15], can be assumed to reduce the transition energy by less than 3 meV between 100 K and 300 K, which is not sufficient to explain the observed behavior.

Second, the influence of the temperature on the effective mass may also be considered altering the confinement energy, although the idea of an effective mass is approaching its limit in structures containing only 26 atoms. Since the effective mass in CdSe quantum dots is expected to decrease slightly[4,6,16] with temperature, this would imply an increase of the confinement energy with increasing temperature, i.e. a reduced red shift with temperature in MSC compared to bulk, in contrast to our findings.

Last, due to the strong confinement of the electron and hole wave functions their overlap and therefore the Coulomb energy scaling with $\frac{1}{r}$ is enhanced[17]. As in a semiconductor the dielectric constant varies with temperature (from 9.17 to 9.64 between 100 and 300 K in CdSe[18]) the Coulomb energy is expected to decrease by about 5% in this temperature range, also leading to a reduced redshift of the bandgap with temperature in MSC compared to bulk, in contrast to the observed behavior.

Supplementary References

1. Efros, A. *et al.* Band-edge exciton in quantum dots of semiconductors with a degenerate valence band: Dark and bright exciton states. *Phys. Rev. B* **54**, 4843–4856 (1996).

2. Yang, J. *et al.* Route to the Smallest Doped Semiconductor: Mn+2-Doped $(CdSe)_{13}$ Clusters. *J. Am. Chem. Soc.* **137**, 12776–12779 (2015).

3. Joshi, A., Narsingi, K. Y., Manasreh, M. O., Davis, E. A. & Weaver, B. D. Temperature dependence of the band gap of colloidal CdSe/ZnS core/shell nanocrystals embedded into an ultraviolet curable resin. *Appl. Phys. Lett.* **89**, 89–92 (2006).

4. Nomura, S. & Kobayashi, T. Exciton–LO-phonon couplings in spherical semiconductor microcrystallites. *Phys. Rev. B* **45**, 1305–1316 (1992).

5. Korsunska, N. E., Dybiec, M., Zhukov, L., Ostapenko, S. & Zhukov, T. Reversible and non-reversible photo-enhanced luminescence in CdSe/ZnS quantum dots. *Semicond. Sci. Technol.* **20**, 876–881 (2005).

6. Liptay, T. J. & Ram, R. J. Temperature dependence of the exciton transition in semiconductor quantum dots. *Appl. Phys. Lett.* **89**, 223132 (2006).

7. Strauch, D. CdSe: phonon dispersion curves, phonon density of states. in *New Data and Updates for several III-V (including mixed crystals) and II-VI Compounds. Landolt-Börnstein - Group III Condensed Matter (Numerical Data and Functional Relationships in Science and Technology)* (ed. Rössler, U.) 127–128 (Springer Berlin Heidelberg, 2012). doi:10.1007/978-3-642-23415-6_76.

8. Bawendi, M. G., Carroll, P. J., Wilson, W. L. & Brus, L. E. Luminescence properties of CdSe quantum crystallites: Resonance between interior and surface localized states. *J. Chem. Phys.* **96**, 946–954 (1992).

9. Landes, C. F., Braun, M. & El-Sayed, M. A. On the Nanoparticle to Molecular Size Transition: Fluorescence Quenching Studies. *J. Phys. Chem. B* **105**, 10554–10558 (2001).

10. Puzder, A., Williamson, A. J., Gygi, F. & Galli, G. Self-healing of CdSe nanocrystals: First-principles calculations. *Phys. Rev. Lett.* **92**, 1–4 (2004).

11. Vörös, M., Galli, G. & Zimanyi, G. T. Colloidal Nanoparticles for Intermediate Band Solar Cells. *ACS Nano* **9**, 6882–6890 (2015).

12. Hernández-Calderón, I. Optical properties and electronic structure of wide band gap II-VI semiconductors. in *II-VI Semiconductor Materials and their Applications* (ed. Tamargo, M. C.) (Taylor and Francis, 2002).

13. Varshni, Y. P. Temperature dependence of the energy gap in semiconductors. *Physica* **34**, 149–154 (1967).

14. Olkhovets, A., Hsu, R.-C., Lipovskii, A. & Wise, F. Size-Dependent Temperature Variation of the Energy Gap in Lead-Salt Quantum Dots. *Phys. Rev. Lett.* **81**, 3539–3542 (1998).

15. Madelung, O. *Semiconductors: Data Handbook*. (Springer-Verlag).

16. Stradling, R. A. & Wood, R. A. The temperature dependence of the band-edge effective masses of InSb, InAs and GaAs as deduced from magnetophonon magnetoresistance measurements. *J. Phys. C Solid State Phys.* **3**, L94–L99 (1970).

17. Brus, L. Electronic wave functions in semiconductor clusters: experiment and theory. *J. Phys. Chem.* **90**, 2555–2560 (1986).

18. Madelung, O., Rössler, U. & Schulz, M. *II-VI and I-VII Compounds; Semimagnetic Compounds*. vol. 41B (Springer-Verlag, Berlin/Heidelberg, 1999).
